# Influence of parotid lymph node metastasis on distant metastasis in parotid gland cancer

**DOI:** 10.3389/fonc.2023.1244194

**Published:** 2023-12-18

**Authors:** Fan Meng, Junhui Yuan, Xu Zhang, Jun Liu, Hailiang Li

**Affiliations:** ^1^ Department of Radiology, The Affiliated Cancer Hospital of Zhengzhou University & Henan Cancer Hospital, Zhengzhou, China; ^2^ Department of Head and Neck, The Affiliated Cancer Hospital of Zhengzhou University & Henan Cancer Hospital, Zhengzhou, China; ^3^ Intensive Care Unit (ICU), Jingzhou Traditional Chinese Medicine Hospital, Jingzhou, Hubei, China

**Keywords:** parotid cancer, distant metastasis, parotid lymph node metastasis, extranodal extension, survival

## Abstract

**Background:**

The aim of this study was to analyze the impact of the number and extranodal extension (ENE) of positive parotid lymph nodes (LNs) on distant metastasis in parotid cancer.

**Methods:**

Patients with surgically treated parotid cancer were retrospectively enrolled. The hazard ratios (HRs) of the number and ENE of positive parotid LNs on distant metastasis-free survival (DMFS) were evaluated.

**Results:**

In the Cox model, the groups with zero and one positive LN had comparable 10-year DMFS, but those with two positive LNs had an HR of 2.11 (95% CI: 1.36–5.29), and those with three or more positive LNs had an HR of 3.31 (95% CI: 2.05–8.43). The presence of ENE in parotid LNs did not impact the DMFS (*p* = 0.462; HR: 2.17; 95% CI: 0.84–6.17).

**Conclusion:**

Parotid LN metastasis was associated with decreased DMFS; this effect was mainly driven by the number of positive LNs rather than ENE.

## Introduction

Parotid cancer is relatively uncommon, and accounts for less than 3% of all head and neck cancers (1). Both surgery and radiotherapy play an essential role in treatment, and the patient’s long-term survival is likely determined mainly by the presence or absence of distant metastasis (DM) rather than locoregional control (2, 3). As a result, the evaluation of potential predictors for DM carries immense significance in improving oncologic outcomes.

Parotid gland is the only major salivary gland to contain lymph nodes (LNs) owing to its development (4). This unique feature offers parotid cancer a pathway of lymphatic drainage, in which parotid LN is the first echelon, and cervical LN is the second echelon (5). Parotid LN metastasis occurs in approximately 20% of parotid cancer (6), and the rate is impacted by tumor size, histologic grade, and other pathologic features (1–3).The importance of neck LN status is recognized in parotid cancer, and an advanced N classification is related to increased risk of DM (7). Officially, N classification is formulated based on the number, size, laterality, and extranodal extension (ENE) of metastatic cervical LNs (8). However, the association between parotid LNs and DM in parotid cancer has rarely been analyzed and we could only deduce from prior literature that parotid LN metastasis provides additional risk of DM (2, 9). Nevertheless, the association of the quantitative burden and of eventual ENE of metastatic parotid LNs on DM development remains unknown. Therefore, our goal was to analyze the impact of the number and ENE of positive parotid LNs on DM in parotid cancer.

## Patients and methods

### Ethical consideration

This study was approved by the Henan Cancer Hospital Institutional Research Committee, and written consent agreements for medical research were obtained from all patients before the initial treatment.

### Study design

To address the purpose, the investigators performed a retrospective study. Medical records of adult patients (>18 years of age) with surgically treated parotid cancer were reviewed between January 1995 and January 2022. The inclusion criteria were as follows: primary epithelial disease; prophylactic or therapeutic neck dissection was conducted and the number of dissected LNs was not smaller than 10; pathologic sections were available for reviewing; total parotidectomy had been performed. Final follow-up data were collected between June 2022 and January 2023 mainly through outpatient records and telephone interviews. Patients with a history of another malignancy, no follow-up data, or distant metastasis at initial treatment were excluded from the study. Information on demography, pathology, treatment, and follow-up of enrolled patients was extracted.

### Study variables

Parotid LN referred to the LN located within the gland. Pathologic tumor and neck LN classification was defined based on the 8th AJCC stage, and pathologic grade was classified into low, intermediate, and high based on the 5th version of the WHO classification (1, 10). Perineural invasion (PNI) was defined as the presence of tumor cells within the nerve, lymphovascular invasion (LVI) was defined as the presence of tumor cells within the lymphovascular vessels, and ENE was defined as the presence of tumor cells outside the capsule of the metastatic LNs. Margin was defined as positive if there were tumor cells involved with the margin in postoperative pathologic analysis.

The primary outcome was distant metastasis-free survival (DMFS), which was confirmed by biopsy or image analysis, if biopsy could not be performed during follow-up, and its time was calculated from the date of surgery to the date of DM detection or last follow-up. The secondary outcome variables were overall survival (OS) and presence of factors (number and ENE) of metastatic parotid LNs. The time of OS was calculated from the date of surgery to the date of last follow-up or death by any cause.

### Treatment principle

All patients underwent surgical treatment under general anesthesia and routine intraoperative pathologic analyses of primary sites were conducted. Total parotidectomy was carried out if there was suspicion of a malignant tumor via frozen section. It was usually performed via a piecemeal or partial approach (11). Prophylactic neck dissection (level I–III) was performed if there was advanced stage disease or other adverse pathologic features, and therapeutic neck dissection (level I–IV/V) was performed if there were clinically or pathologically positive cervical LNs. Adjuvant radiotherapy or chemotherapy was suggested for patients with a T3/4 tumor, LN metastasis, positive margin, PNI, or LVI. After treatment completion, each patient was followed up every 3–6 months in the first 2 years, every 6–12 months in the next 3 years, and every 1–2 years thereafter.

For patients with only locoregional recurrence, a salvage operation was usually the first choice if possible, whereas for patients with DM, palliative treatment tended to be given.

### Statistical analysis

Missing data patterns among tumor classification, neck classification, pathologic grade, PNI, and LVI were deemed not missing completely at random (12). Missing rates among the variables were 18.8% for grade, 13.7% for tumor classification, 12.0% for PNI, 11.2% for LVI, and 10.0% for neck classification. Missing data were imputed using multiple imputation using Fully Conditional Specifications implemented by the multiple imputation by chained equations algorithm (13).

Factors that were significant in univariate Cox analyses were then analyzed in multivariate Cox proportional hazard regression analyses to determine the independent variables for DMFS and OS. The association between factors of metastatic parotid LNs and clinicopathological variables was evaluated using the Chi-square test. All analyses were performed using R software 3.4.3, and a *p*-value less than 0.05 was considered statistically significant.

## Results

### Baseline data

In total, 490 patients were included, with 235 (48.0%) male and 255 (52.0%) female patients, and the mean age was 53 ± 21 years. Preservation of facial nerve was successful in 357 (72.9%) patients. Pathologic tumor classification was defined as T1 in 67 (13.7%) patients, T2 in 133 (27.1%) patients, T3 in 192 (39.2%) patients, and T4 in 98 (20.0%) patients. Pathologic N status was defined as N0 in 267 (54.5%) patients, N1 in 120 (24.5%) patients, N2 in 63 (12.9%) patients, and N3 in 40 (8.2%) patients. ENE of cervical LNs occurred in 46 (20.6%, 46/223) patients. Furthermore, pathologic grade was low in 100 (20.4%) patients, intermediate in 275 (56.1%) patients, and high in 115 (23.5%) patients (**Supplementary Table 1**). PNI and LVI developed in 96 (19.6%) and 77 (15.7%) patients, respectively. All patients underwent a total parotidectomy and a positive margin was noted in 30 (6.1%) patients. Adjuvant radiotherapy was performed in 287 patients, of whom 85 patients also received chemotherapy.

There was parotid LN metastasis in 198 (40.4%) patients, out of whom 89 patients had one positive LN, 54 had two positive LNs, and 55 had three or more positive LNs. ENE occurred in 39 patients with metastatic LNs.

### Predictor for DMFS

After a follow-up with a mean time of 5.4 ± 2.8 years, DM developed in 160 patients with a mean time of 3.0 ± 1.5 years after initial treatment. Lung-only metastasis occurred in 100 patients. In the remaining 60 patients, metastasis occurred in the lungs in 30 patients, the bone in 18 patients, the liver in 17 patients, and the brain in 10 patients. The overall 10-year DMFS rate was 58% [95% confidence interval (CI): 52%–64%].

**Figure 1:**
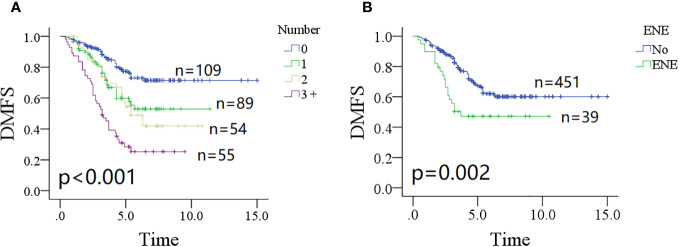
Comparison of distant metastasis-free survival (DMFS) in patients with different number of positive lymph nodes (**A**, *p* < 0.001) and extranodal extension (**B**, *p* = 0.002).

The 10-year DMFS rates were 71% (95% CI: 65%–77%) for patients without parotid LN metastasis, 52% (95% CI: 38%–66%) for patients with one positive parotid LN, and 41% (95% CI: 25%–57%) for patients with two positive parotid LNs. In patients with three or more metastatic parotid LNs, the 5-year DMFS rate was 28% (95% CI: 16%–40%). The difference was significant (**Figure 1A**, *p* < 0.001). Patients without ENE in parotid LNs had a 10-year DMFS rate of 59% (95% CI: 53%–65%), which was significantly higher than in patients with ENE in parotid LNs [47% (95% CI: 31%–63%)] (**Figure 1B**, *p* = 0.002). Other significant factors included tumor classification, neck status, pathologic grade, PNI, and positive margin (all *p* < 0.05, **Table 1**).

**Table 1:** Univariate and multivariate Cox model analyses of predictors for distant metastasis-free survival.

Variable	Univariable	Multivariable	
	*p*	*p*	HR [95% CI]
Age			
≤50			
>50	0.487		
Sex			
Male			
Female	0.732		
Preservation of facial nerve	0.364		
Pathologic tumor classification			
T1			Ref
T2		0.276	1.43 [0.67–4.19]
T3		0.012	2.31 [1.56–6.52]
T4	<0.001	<0.001	3.19 [2.01–10.88]
Pathologic neck classification			
N0			Ref
N1		0.370	1.95 [0.73–4.85]
N2		<0.001	2.11 [1.72–7.11]
N3	<0.001	<0.001	3.56 [2.23–12.32]
Pathologic grade			
Low			Ref
Intermediate		0.033	1.84 [1.25–3.96]
High	<0.001	<0.001	3.96 [2.31–11.76]
PNI^%^			
No			Ref
Yes	<0.001	0.454	2.07 [0.47–7.17]
LVI^^^			
No			
Yes	0.142		
Positive margin			
No			Ref
Yes	<0.001	<0.001	5.27 [2.14–18.99]
Parotid LN metastasis^*^			
0			Ref
1		0.160	1.74 [0.82–4.22]
2		0.006	2.11 [1.36–5.29]
3+	<0.001	<0.001	3.31 [2.05–8.43]
ENE of parotid LN^&^			
No			Ref
Yes	0.002	0.462	2.17 [0.84–6.17]
Treatment^!^			
S			
S+R			
S+R+C	0.117		

% PNI, Perineural invasion;

^ LVI, lymphovascular invasion;

* LN, lymph node;

& ENE, extranodal extension;

! S, surgery; R, radiotherapy; C, chemotherapy.

In the Cox model, compared with non-metastatic parotid LNs, one positive LN did not compromise the DMFS, but two positive LNs were associated with increased risk of DM [*p* = 0.006, hazard ratio (HR): 2.11, 95% CI: 1.36–5.29], and three or more positive LNs had an HR of 3.31 (95% CI: 2.05–8.43). Presence of ENE in parotid LNs was not related to the additional possibility of DM compared with the non-ENE group (*p* = 0.462). Positive margin had the greatest HR of 5.27 (95% CI: 2.14–18.99). Presence of a T3 or T4 classification was associated with increased 1.5- or 2.0-fold risk of DM. N2 and N3 classifications had an HR of 2.11 (95% CI: 1.72–7.11) and 3.56 (95% CI: 2.23–12.32), respectively. Compared with low grade, intermediate (HR: 1.84; 95% CI: 1.25–3.96) and high (HR: 3.96; 95% CI: 2.31–11.76) grades had a higher possibility of DM occurrence (**Table 1**).

### Subgroup analysis

**Figure 2:**
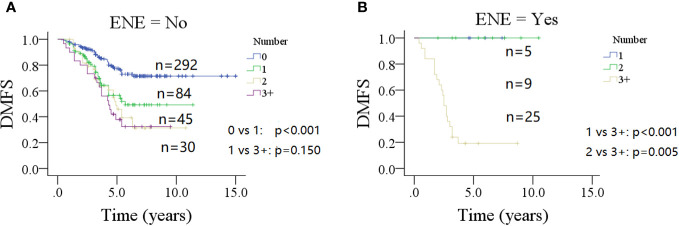
Comparison of distant metastasis-free survival (DMFS) in patients with different number of positive lymph nodes stratified by the status of extranodal extension (ENE) of parotid lymph node (**A** for no ENE and **B** for ENE).

In a subgroup of patients with no ENE of parotid LNs (**Figure 2A**), the 10-year DMFS rate was 49% (95% CI: 35%–63%) in patients with one metastatic parotid LN, which was significantly lower than in patients with no parotid LN metastasis 71% (95% CI: 65%–77%, *p* < 0.001), but comparable to those with two or three or more positive parotid LNs (*p* = 0.201 and *p* = 0.150, respectively).

In a subgroup of patients with ENE of parotid LNs (**Figure 2B**), the 5-year DMFS rates were 100% for patients with one or two positive parotid LNs, but only 19% (95% CI: 3%–35%) in patients with three positive or more metastatic parotid LNs (*p* < 0.001 and *p* = 0.005, respectively).

### Predictor for OS

During our follow-up, death occurred in 135 patients, the causes included cancer recurrence (*n* = 109), systemic disease (*n* = 20), secondary cancer (*n* = 4), and traffic accident (*n* = 2). The 10-year OS rate was 52% (95% CI: 44%–60%).

**Figure 3:**
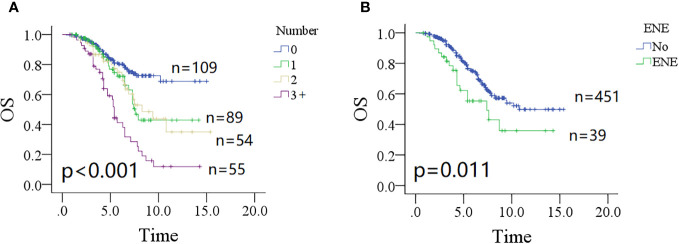
Comparison of overall survival (OS) in patients with different number of positive lymph nodes (**A**, *p* < 0.001) and extranodal extension (**B**, *p* = 0.011).

The 10-year OS rates were 73% (95% CI: 65%–81%) for patients without parotid LN metastasis, 43% (95% CI: 27%–59%) for patients with one positive parotid LN, 43% (95% CI: 25%–61%) for patients with two positive parotid LNs, and 12% (95% CI: 0%–24%) for patients with three or more positive parotid LNs; the difference was significant (**Figure 3A**, *p* < 0.001). Patients without ENE in parotid LNs had a 10-year OS rate of 54% (95% CI: 46%–62%), which was significantly higher than in patients with ENE in parotid LNs [35% (95% CI: 13%–57%)] (**Figure 3B**, *p* = 0.011). Other significant factors included tumor classification, neck status, pathologic grade, and positive margin (all *p* < 0.05, **Table 2**).

**Table 2:** Univariate and multivariate Cox model analyses of predictors for overall survival.

Variable	Univariable	Multivariable	
	*p*	*p*	HR [95% CI]
Age			
≤50			
>50	0.178		
Sex			
Male			
Female	0.297		
Preservation of facial nerve	0.668		
Pathologic tumor classification			
T1			Ref
T2		0.337	1.98 [0.76–4.27]
T3		<0.001	2.37 [1.26–7.45]
T4	<0.001	<0.001	4.18 [2.11–17.53]
Pathologic neck classification			
N0			Ref
N1		0.240	1.67 [0.84–3.14]
N2		<0.001	2.35 [1.87–5.26]
N3	<0.001	<0.001	3.05 [2.00–7.18]
Pathologic grade			
Low			Ref
Intermediate		0.240	1.90 [0.77–4.44]
High	<0.001	<0.001	3.29 [1.90–8.27]
PNI^%^			
No			
Yes	0.527		
LVI^^^			
No			
Yes	0.338		
Positive margin			
No			Ref
Yes	<0.001	<0.001	5.28 [2.17–18.26]
Parotid LN metastasis^*^			
0			Ref
1		0.240	1.56 [0.83–3.26]
2		0.061	1.88 [0.98–3.86]
3+	<0.001	<0.001	2.86 [1.75–6.27]
ENE of parotid LN^&^			
No			Ref
Yes	0.011	0.337	2.06 [0.74–6.15]
Treatment^!^			
S			
S+R			
S+R+C	0.225		

% PNI, Perineural invasion;

^ LVI, lymphovascular invasion;

* LN, lymph node;

& ENE, extranodal extension;

! S, surgery; R, radiotherapy; C, chemotherapy.

In the Cox model, both non-metastatic and one metastatic parotid LN groups had comparable mortality possibility (*p* = 0.240); however, groups with two or more positive LNs had an additional possibility of death with an HR of 1.88 (95% CI: 0.98–3.86) for two positive metastatic LNs, and 2.86 (95% CI: 1.75–6.27) for three or more positive LNs. Compared with non-ENE, ENE did not provide an extra overall risk of death (*p* = 0.337). Tumor classification of T3/4, neck status of N2/3, and high pathologic grade significantly increased the overall death risk, and the impact of positive margin was the greatest with an HR of 5.28 (95% CI: 2.17–18.26) (**Table 2**).

### Subgroup analysis

**Figure 4:**
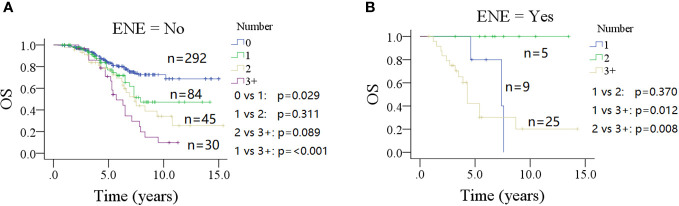
Comparison of overall survival (OS) in patients with different number of positive lymph nodes stratified by the status of extranodal extension (ENE) of parotid lymph node (**A** for no ENE and **B** for ENE).

In a subgroup of patients with no ENE of parotid LNs (**Figure 4A**), the 10-year OS rate was 48% (95% CI: 30%–64%) for patients with one positive parotid LN, which was inferior to the OS rate in patients without parotid LN metastasis (*p* = 0.029) and superior to those with three or more positive parotid LNs [10% (95% CI: 0%–22%)] (*p* < 0.001). However, this was similar to patients with two metastatic parotid LNs [33% (95% CI: 15%–51%)] (*p* = 0.311). Groups with two or three or more positive metastatic parotid LNs had analogous 10-year OS rates (*p* = 0.089).

In a subgroup of patients with ENE of parotid LN (**Figure 4B**), the 5-year OS rates were 78% (95% CI: 38%–100%) for one positive parotid LN, and 100% for two positive LNs; the difference was not significant (*p* = 0.370). However, both of them were higher than that seen in the three or more positive metastatic parotid LNs group [43% (95% CI: 21%–65%)] (*p* = 0.012 and *p* = 0.008, respectively).

### Relationship between metastatic parotid LNs and clinicopathological variables

Parotid LN metastasis was associated with tumor classification (*p* < 0.001), N status (*p* < 0.001), and pathologic grade (*p* < 0.001), but showed little relationship with age (*p* = 0.955), sex (*p* = 0.696), PNI (*p* = 0.867), LVI (*p* = 0.610), ENE of parotid LNs (*p* = 0.537), or cervical LNs (*p* = 0.513). Patients with T3/4, N2/3, and high pathologic grade were likely to develop multiple metastatic parotid LNs (**Table 3**). The only factor related to ENE of parotid LNs was pathologic grade (*p* = 0.002), especially high pathologic grade, which meant an increased possibility of 15.7% for ENE occurrence. Other clinicopathological variables did not seem to impose any effect on ENE of parotid LNs, including tumor classification (*p* = 0.949), N classification (*p* = 0.993), PNI (*p* = 0.880), and LVI (*p* = 0.391) (**Table 4**).

**Table 3:** Association between the number of metastatic parotid lymph nodes (LNs) and clinicopathologic variables.

Variable	Number of metastatic parotid lymph nodes				*p*
	0 (*n* = 292)	1 (*n* = 89)	2 (*n* = 54)	3+ (*n* = 55)	
Age					
≤50	156	46	27	30	
>50	136	43	27	25	0.955
Sex					
Male	145	43	24	23	
Female	147	46	30	32	0.696
Pathologic tumor classification					
T1	56	6	3	2	
T2	116	8	5	4	
T3	98	36	27	31	
T4	22	39	19	18	<0.001
Pathologic neck classification					
N0	222	34	6	5	
N1	69	30	11	10	
N2	1	21	20	21	
N3	0	4	17	19	<0.001
Pathologic grade					
Low	79	12	4	5	
Intermediate	179	47	24	25	
High	24	40	26	25	<0.001
PNI^%^					
No	242	69	44	44	
Yes	55	20	10	11	0.867
LVI^^^					
No	248	71	47	47	
Yes	44	18	7	8	0.610
ENE of parotid LN^&^					
No	269	79	51	52	
Yes	23	10	3	3	0.537
ENE of cervical LN					
No	266	77	50	51	
Yes	26	12	4	4	0.513

% PNI, Perineural invasion;

^ LVI, lymphovascular invasion;

& ENE, extranodal extension.

**Table 4:** Association between extranodal extension (ENE) of metastatic parotid lymph nodes (LNs) and clinicopathologic variables.

Variable	ENE of metastatic parotid LN		*p*
	No (*n* = 451)	Yes (*n* = 39)	
Age			
≤50	237	22	
>50	214	17	0.643
Sex			
Male	220	15	
Female	231	24	0.216
Pathologic tumor classification			
T1	62	5	
T2	122	11	
T3	178	14	
T4	89	9	0.949
Pathologic neck classification			
N0	245	22	
N1	111	9	
N2	58	5	
N3	37	3	0.993
Pathologic grade			
Low	93	7	
Intermediate	261	14	
High	97	18	0.002
PNI^%^			
No	363	31	
Yes	88	8	0.880
LVI^^^			
No	382	31	
Yes	69	8	0.391
ENE of cervical LN			
No	411	33	
Yes	40	6	0.244

% PNI, Perineural invasion;

^ LVI, lymphovascular invasion.

## Discussion

The most important finding in our study was that parotid LN metastasis significantly affected the DM possibility and OS in parotid cancer. Furthermore, this influence was mainly based on the number of metastatic LNs. After accounting for the number of metastatic LNs, ENE of parotid LNs was no longer related to DMFS or OS, but impacted the action pattern of the number of metastatic LNs.

DM was the main explanation for death in salivary gland cancer, and extensive literature had explored the predictors of DM (7, 14–16). Nam et al. (7) enrolled 454 patients with previously untreated salivary gland cancer, and noted that 20.9% of the sample developed DM. Multivariate analysis showed that high grade, non-parotid origin, PNI, T3/4 classification, and N2/3 classification were independent variables for DMFS. Furthermore, in a study consisting of 418 patients who had undergone surgery followed by radiotherapy (14), the 10-year DMFS rate was 59.1%, and independent factors included age, high pathologic grade, advanced T classification, positive N status, and high platelet-to-lymphocyte ratio. The nomogram based on these variables had a satisfactory individual prediction for DMFS. Another report identified 884 patients (14), and 15% of the population suffered from DM after initial treatment; the factors remaining significant in predicting shorter DMFS were male sex, advanced T or N classifications, and high pathologic grade. Similar findings were described by Lim et al. (16) and Yan et al. (17). Our study would also support the effect of common adverse pathologic variables on DM; however, the above-mentioned studies neglected the impact of metastatic parotid LNs, and did not analyze this variable in multivariate analyses. Nevertheless, an agreement of survival compromise added by parotid LN metastasis has been widely reached (18, 19), but to the best of our knowledge, only one previous research has evaluated the association between metastatic parotid LNs and DM. In this study (3), parotid LN metastasis occurred in 31.8% of the 144 patients with parotid cancer, and it was related to an additional onefold risk of DM compared with the non-metastasis group. It revealed that parotid LN metastasis could increase the risk of DM and our study provided deeper insight into the relationship. The DMFS and OS were the same between non-metastatic and one positive LN groups, but significantly decreased if there were two or more metastatic LNs. This finding was of great significance, as, on one hand, survival rate could be decreased by up to half if metastasis developed in only one LN in head and neck squamous cell carcinoma (20). This may be explained by the distinct difference of biologic behavior between the two kinds of disease, parotid LN metastasis was frequent in parotid cancer, and the common pattern was one metastatic LN, which was also an indicator for adjuvant therapy. Our finding may benefit in adjusting treatment plans for patients to improve their quality of life without downgrading survival.

ENE was an important factor of metastatic LNs; it predicted a poorer prognosis in salivary gland cancer and was a strong indicator for adjuvant chemotherapy to decrease the possibility of DM, which was also taken into consideration during LN status determination (21). The outcome in 114 patients with pN+ salivary gland carcinoma was analyzed by Hsieh et al. (22). ENE developed in 51% of the population, and was related to advanced N classification, PNI, higher number of positive LNs, and LVI, but had no association with demography, histology grade, tumor classification, or tumor origin. Moreover, after adjusting for the number of positive cervical LNs, there was little influence on prognosis by ENE. In another study performed by Qian et al. (23), ENE occurred in 27 (40.9%) of patients, and this population had comparable locoregional-free survival, DMFS, and OS with patients without ENE. Although the finding of the two studies was based on the ENE of cervical LNs, it elucidated that ENE in salivary gland cancer demonstrated no influence on survival, but was correlated directly with adverse pathological features that influenced prognosis (24). Whether this principle applied to parotid LNs remained unknown. Lombardi et al. (25) may be the only ones to report the effect of ENE in parotid LN on survival. In their study, 89 patients with pathologically positive neck classification were analyzed, of whom 55 had ENE in parotid LNs and 34 did not. The two groups had similar OS, a finding that is supported by our analysis. More interestingly, the current study reported no supernumerary DMFS deterioration by ENE of parotid LNs. The underlying mechanism may be due to the small anatomical size of the parotid LNs; even a minimal lesion can easily break through the capsule. This finding could reduce the administration of chemotherapy to optimize the adjuvant therapy project. Additionally, our study demonstrated the action pattern of the number of metastatic parotid LNs that could be altered by ENE of parotid LNs. The effect of parotid LN began to occur when there was one positive LN under the circulation of no-ENE, but began to occur when there were at least three positive LNs in the presence of ENE. This discovery may be due to the fact that intermediate- or high-grade disease has a higher risk of ENE presence and multiple metastatic LNs; hence, the survival compromise of the subgroup was likely driven by these adverse pathologic features.

A pooled prevalence of parotid LN metastasis in the unselected studies was 24.1% according to a review, and its association with clinicopathological variables has been widely analyzed and the common risk factors included advanced stage and pathologic grade (3, 18). Our study not only confirmed these conclusions, but also uncovered that multiple metastatic parotid LNs were likely to develop when there was the presence of T3/4, N2/3, and high pathologic grade parotid cancer. We also noted that the only factor affecting the ENE of parotid LNs was pathologic grade, which was an inherent feature of high-grade disease.

This study had several limitations. First, the retrospective nature of the study meant that it had inherent bias, which may have decreased its statistical power. Second, this was a single institution study, and our findings require validation of external data and a prospective research before they can be applied. Third, further follow-up was needed for more interesting findings.

In conclusion, distant metastasis in parotid cancer was common and likely to occur in one-third of the population within 3 years after initial treatment. We found that parotid LN metastasis was associated with DMFS and OS, and the effect was mainly driven by the quantity of affected LNs and influenced by ENE. Our study may benefit in optimizing patient’s adjuvant therapy plans by reducing unnecessary treatments.

## Data availability statement

The original contributions presented in the study are included in the article/**Supplementary Material**. Further inquiries can be directed to the corresponding author.

## Ethics statement

This study was approved by Henan Cancer Hospital Institutional Research Committee. The studies were conducted in accordance with the local legislation and institutional requirements. The participants provided their written informed consent to participate in this study.

## Author contributions

All authors listed have made a substantial, direct, and intellectual contribution to the work, and approved it for publication.
