# Supplementary material for: Influence of parotid lymph node metastasis on distant metastasis in parotid gland cancer
Source: Front Oncol. 2023 Dec 18;13:1244194. doi: 10.3389/fonc.2023.1244194 (PMC10759210; doi:10.3389/fonc.2023.1244194)
Supplement: Supplementary file 1 [file Table_1.doc]

Supplementary Table 1. Pathologic type of the enrolled patients.

| Cancer type | N |
| --- | --- |
| High grade (n=115, 23.5%) |  |
| Mucoepidermoid carcinoma | 50 |
| Duct carcinoma | 28 |
| Adenocarcinoma not otherwise specified | 15 |
| Spindle cell carcinoma | 12 |
| Large/small cell carcinoma | 10 |
| Moderate grade (n=275, 56.1%) |  |
| Mucoepidermoid carcinoma | 114 |
| Adenoid cystic carcinoma | 87 |
| Myoepithelial carcinoma | 74 |
| Low grade (n=100, 20.4%) |  |
| Mucoepidermoid carcinoma | 32 |
| Acinic cell carcinoma | 21 |
| Pleomorphic low-grade adenocarcinoma | 19 |
| Basal cell carcinoma | 17 |
| Epithelial-myoepithelial carcinoma | 11 |
